# Acute Aortic Dissection in Pregnancy in a Woman with Undiagnosed Marfan Syndrome

**DOI:** 10.1155/2012/490169

**Published:** 2012-12-13

**Authors:** Mandana Master, Gavin Day

**Affiliations:** ^1^Department of Obstetrics and Gynaecology, Women's and Children's Hospital, Adelaide, SA 5006, Australia; ^2^Department of Cardiothoracic Surgery, Flinders Medical Centre, Adelaide, SA 5142, Australia

## Abstract

We report a case of acute aortic dissection in a lady of 28 weeks of gestation with undiagnosed Marfan syndrome. The patient had been seen in our antenatal clinics. Her history documented in her pregnancy record was negative for genetic/congenital abnormalities. There was no family history documented. Subsequently, at 28 weeks of gestation, the patient presented with sudden onset chest, jaw, and back pain. Further history revealed that her father had died at the age of 27 of an aortic dissection. Echocardiography showed aortic root dissection with occlusion of aortic branches. She subsequently underwent an emergency lower segment caesarean section followed by surgical repair of type A dissection. A simultaneous type B dissection was managed conservatively. On later examination, our patient fulfilled the diagnostic criteria for phenotypic expression of Marfan syndrome. Genetic testing also confirmed that she has a mutation of the fibrillin (FBN 1) gene associated with the disease.

## 1. Background

Marfan syndrome is an autosomal dominant inherited connective tissue disorder with an estimated prevalence of 2-3 in 10 000 [1]. The primary manifestations of Marfan syndrome are musculoskeletal, ocular, and cardiovascular. The cardiovascular features include mitral valve prolapse, mitral regurgitation, aortic root dilatation, and aortic incompetence. Acute aortic dissection and rupture of the aorta carry a high risk of maternal mortality, and if prepartum, fetal demise. 

During pregnancy, important maternal cardiovascular changes occur including an increase in blood volume, heart rate, cardiac output, left ventricular wall mass, and end diastolic dimensions. In addition, hormonal changes accelerate the development of pathologic changes in the arterial wall. Fragmentation of the reticulum fibres, a diminished amount of acid mucopolysaccharides and loss of the normal corrugation of elastic fibres have been observed in the aortic wall of pregnant patients. Therefore, both haemodynamic and hormonal mechanisms increase the susceptibility to dissection in pregnant women [2]. 

Recent medical literature has suggested that the risk of complications is less than 1% in women with minimal cardiovascular involvement and root diameter < 40 mm and up to 10% in those with ascending aorta > 40 mm [3]. However, there is still some debate regarding the aortic root diameter above which pregnancy should be discouraged in women with Marfan syndrome [4]. Nevertheless, appropriate counselling and close echocardiographic followup cannot be applied if women have not been diagnosed prior to pregnancy. A missed diagnosis may result in a potentially life-threatening presentation to hospital, as described in this paper.

## 2. Case Presentation

A 27-year-old G4P0 lady of 28 of weeks gestation presented with sudden onset of chest pain, radiating to her jaw and back. Her vital signs were within normal limits, electrocardiogram showed normal sinus rhythm and her blood results were unremarkable, in particular cardiac enzymes were not elevated. She reported reduced fetal movements and was therefore transferred to a nearby maternity hospital.

Her past obstetric history included stillborn twins of 27 weeks of gestation at the age of 16, which was attributed to considerable antenatal alcohol and drug use. Later in her early twenties she had two terminations.

Antenatal care for this pregnancy was commenced with a general practitioner and was then referred to a midwifery practice at her request. At 20 weeks of gestation, morphology ultrasound showed a right hydronephrotic kidney in the fetus and she was referred for antenatal care at a tertiary hospital.

At the maternity hospital, she remained haemodynamically stable but she was found to have a faint indeterminate murmur and Troponin T was elevated to 0.04 (normal range < 0.02 *μ*g/L). There was no radio-radio delay or radio-femoral delay bilaterally and she reported no shortness of breath. She complained of ongoing chest and back pain, which settled with analgesia. Cardiotocography (CTG) was normal but she was given corticosteroids in case of preterm birth. 

Overnight, the patient went into cardiogenic shock. Chest X-ray showed cardiomegaly, acute pulmonary oedema and a prominent descending aorta. An urgent transthoracic echocardiogram revealed a 6 cm aortic root dissection with occlusion of two out of three aortic branches.

**Figure 1:**
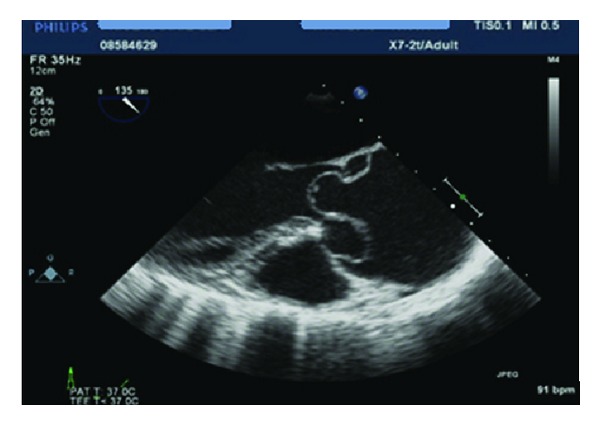
Transoesophageal echocardiogram showing Type A aortic dissection with effacement of the sinotubular junction.

**Figure 2:**
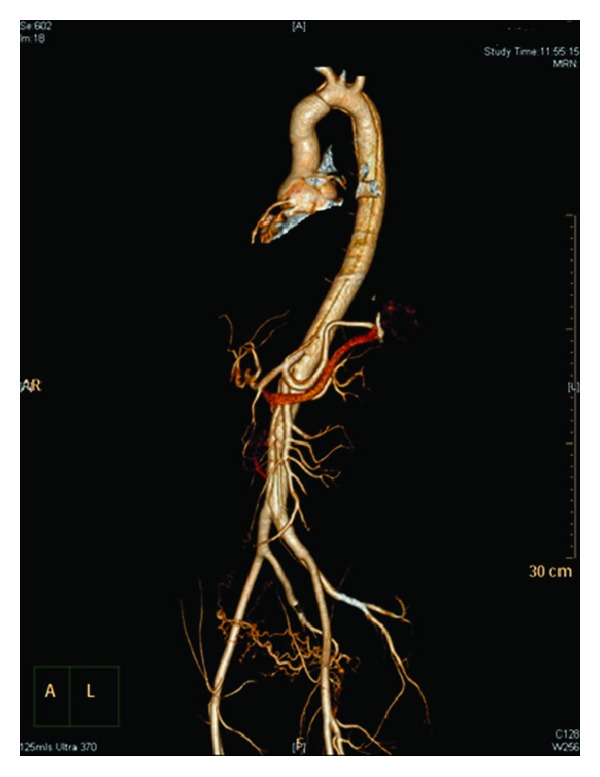
CT 3D reconstruction showing aortic dissection in the arch of the aorta extending along the descending thoracic aorta. Distally the dissection extends along the abdominal aorta up to the aortic bifurcations into the left common iliac artery.

The patient was resuscitated and transferred to the intensive care unit (ICU) of another tertiary hospital. She had an emergency lower segment caesarean section under general anaesthesia and a live male infant (1610 g) was delivered without complication. The neonate was transferred to the ICU for management of extreme prematurity. The cardiothoracic team then commenced repair of the aortic dissection. Intraoperative transoesophageal echo indicated the presence of simultaneous type A and type B aortic dissections (as shown in Figure 1). The decision was made to surgically repair type A dissection and to treat type B dissection conservatively (Figure 2). 

Post-op the patient was transferred to the intensive care unit (ICU) in a stable condition. The next day she was found to have bilateral haemothraces and two pigtail drains were inserted. On day two she became symptomatic of acute pulmonary oedema and type 1 respiratory failure and was found to have a right upper lobe pulmonary embolus, which was treated successfully. 

On day 7 post-op, the patient was transferred to the ward. Unfortunately on day 12 she developed a wound infection of the right groin which required treatment with IV antibiotics and multiple debridements in theatre. She was discharged-day 29 post-op.

Whilst on the ward, the patient was identified as having features clinically suggestive of Marfan syndrome. She had the following traits on history and physical exam [5].

### 2.1. Skeletal System

#### 2.1.1. Major Criteria

- A reduced upper to lower segment of 0.78 (versus 0.93 normally).
- Arm span exceeding height, of 179 cm/164 cm, giving a ratio of 1.09 (versus a normal ratio <1.05).
- Arachnodactyly, with positive wrist and thumb signs.
- Pectus carinatum.

#### 2.1.2. Minor Criteria

- Joint hypermobility high-arched palate and crowding of teeth.

### 2.2. Cardiovascular System

#### 2.2.1. Major Criteria

- Dissection of the ascending aorta.

#### 2.2.2. Minor Criteria

- Dissection of the descending thoracic aorta below the age of 50.

### 2.3. Ocular Findings

#### 2.3.1. Minor Criteria

- Myopia.

### 2.4. Other Findings

#### 2.4.1. Minor Criteria

- Extensive cutaneous striae distensae on her legs, back, shoulders, and abdomen.
- She reported bilateral inguinal hernia repairs as a child.

## 3. Outcome and Followup

**Figure 3:**
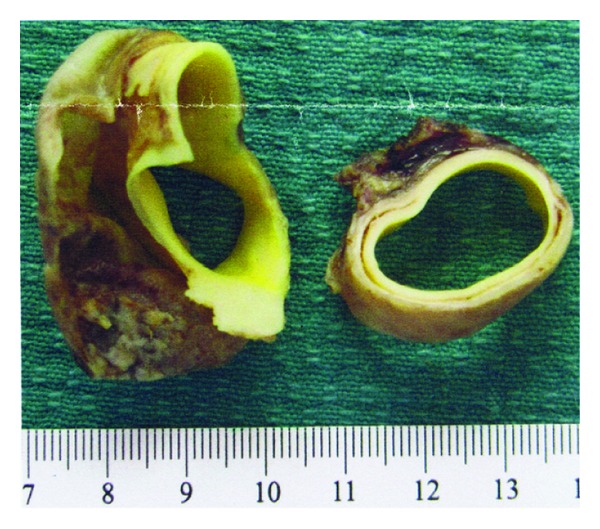
Aortic dissection flap and aortic wall.

Histology of the aortic wall (Figure 3) was suggestive of a collagen abnormality and she was referred to a clinical genetic service for further investigation. She was diagnosed with Marfan disease and genetic testing showed the presence of the FBN 1 mutation associated with the disease. The infant also carries the FBN 1 mutation. He has ongoing cardiology followup for hypertension and borderline high aortic root size but normal valves. He has mild myopia and is on the waiting list for a nephrectomy for an atrophic right kidney.

## 4. Discussion

This patient carried the classical appearance of Marfan syndrome which was identified in retrospect. Although the patient did not indicate during her antenatal visits that her father had passed away from an aortic dissection at the age of 27, her phenotypic appearance was classical. This experience has alerted us to the fact that it is imperative as clinicians, when providing prepregnancy care, to consider genetic conditions such as Marfan syndrome as missing such a diagnosis can lead to complications which are devastating. 

In cases where Marfan syndrome is diagnosed prior to pregnancy, appropriate preconception counselling can take place. Preconception counselling should draw attention to the risk of pregnancy in both the mother and child. Women with Marfan syndrome should be evaluated for cardiovascular abnormalities before pregnancy. Complications occur more commonly in patients with aortic root dilation > 40 mm or rapid progression of the dilation or previous dissection. In most cases, aortic dissection has occurred in women in the third decade of life, therefore it is advisable to plan pregnancy at a younger age. If indicated, elective surgical repair of an enlarged aortic root should be done prepartum as it can be performed with low morbidity and mortality [6].

In instances where the diagnosis is made during pregnancy, low-risk women should have echocardiographic evaluation of the size of the aorta each trimester and prior to delivery. Beta blockers are the mainstay of medical treatment as they have been shown to slow the growth of the aortic root. As beta blockers are category C, they should be used only when the potential benefit outweighs the risk to the fetus. Selective beta blockers such as metoprolol are the preferred choice [7]. Vaginal delivery can be safely performed in these women however in order to reduce the stress of labour, epidural anaesthesia and vacuum or forceps delivery to shorten the second stage are recommended. In high-risk women (aortic root diameter > 40 mm, progressive dilation during pregnancy or previous dissection) elective caesarean section is the preferred mode of delivery [4].
